# Semantic Channel Capacity of Rayleigh Fading Channels Based on Synonymous Mapping

**DOI:** 10.3390/e28060588

**Published:** 2026-05-26

**Authors:** Yuxin Han, Sen Wang, Yaping Sun, Kai Niu, Nan Ma, Ping Zhang

**Affiliations:** 1 Key Laboratory of Universal Wireless Communications, Ministry of Education, Beijing University of Posts and Telecommunications, Beijing 100876, China; hanyx@bupt.edu.cn; 2 Department of Broadband Communication, Pengcheng Laboratory, Shenzhen 518055, China; sunyp@pcl.ac.cn (Y.S.); manan@bupt.edu.cn (N.M.); 3 China Mobile Research Institute, Beijing 100080, China; wangsenyjy@chinamobile.com; 4 Key Laboratory of Networking and Switching Technology, Beijing University of Posts and Telecommunications, Beijing 100876, China; pzhang@bupt.edu.cn

**Keywords:** semantic communication, semantic channel capacity, semantic information theory, Rayleigh fading channel

## Abstract

Classical information theory (CIT) characterizes the transmission limit for communication systems under syntactic accuracy, whereas semantic information theory (SIT) studies communication from the perspective of semantic fidelity induced by synonymous mapping. In this paper, we investigate the semantic channel capacity of Rayleigh fading channels under synonymous mapping of the channel gain and additive noise. We first derive the semantic capacity formula when synonymous mapping is applied to the channel fading coefficient and establish corresponding upper and lower bounds using Jensen’s inequality. To determine an optimized synonymous partition, the partition design is formulated as a constrained optimization problem and solved numerically using a neural network-based approach with the Adam optimizer. Furthermore, we extend the framework by applying synonymous mapping to both the channel fading coefficient and the additive noise and derive the corresponding semantic capacity formula together with its theoretical bounds. The numerical results illustrate the theoretical semantic channel capacity under synonymous mapping and validate the compatibility of the proposed framework with both CIT and SIT. At a 20-dB SNR with K=8 channel gain intervals and J=4 noise intervals, the semantic capacity reached 9.86 sebits/s/Hz.

## 1. Introduction

Since Claude E. Shannon established the mathematical theory of communication in 1948 [1], classical information theory (CIT) has been the theoretical foundation guiding the design and optimization of communication systems. The Shannon capacity formula $C=\max_{p(x)}I\left(X;Y\right)$ provides the fundamental limit on reliable transmission rates over noisy channels. Over the past few decades, advanced coding techniques such as turbo codes [2], LDPC codes [3], and polar codes [4] have successfully approached these theoretical limits. However, CIT does not account for the semantic meaning of transmitted data, treating all information as syntactic symbols (Level A) [5]. Weaver identified two additional levels beyond the technical problem: the semantic problem, concerning how precisely symbols convey desired meaning (Level B), and the effectiveness problem, addressing how effectively meaning affects conduct (Level C).

As wireless communication systems evolve toward 6G and beyond, two factors motivate the paradigm shift toward semantic communication: (1) the traditional bit-oriented approach encounters bottlenecks in spectral efficiency and energy consumption, and (2) in many practical scenarios, the receiver is interested in the semantic content rather than exact syntactic symbols [6,7]. Recent years have witnessed growing research interest in semantic communication [8,9,10,11,12,13,14]. As wireless systems evolve toward 6G [15], many scenarios focus less on reliable bit-level transmission and more on conveying meaning or task-relevant information. This trend exposes the limitations of Shannon’s syntactic view. Deep learning-based approaches have demonstrated impressive performance in transmitting semantic information across multiple modalities, including text [16], speech [17], and images [18,19,20]. In addition, advanced architectures incorporating nonlinear transforms [21] and adaptive channel feedback [22] have further improved end-to-end semantic coding efficiency.

From an information theoretic perspective, early attempts to formalize semantic information include the logical probability framework [6], strongly semantic information theory [7], and the Syntax-Semantics-Pragmatics Trinity model [23,24]. More recently, goal-oriented and task-oriented semantic communication frameworks have characterized information relevance and importance by their utility for specific downstream tasks. Liu et al. [25] derived an indirect rate distortion characterization for semantic sources, and Stavrou and Kountouris [26] investigated the role of fidelity in goal-oriented semantic communication from a rate distortion perspective. Shao et al. [27] further studied the relationship between rate distortion theory and semantic efficiency in task-oriented systems. These works collectively establish that the value of information should be measured by its semantic relevance and task importance, rather than syntactic fidelity alone.

Niu and Zhang recently established a systematic semantic information theory (SIT) [28] by introducing the concept of *synonymous mapping*, which partitions syntactic symbols into semantic equivalence classes and provides a mathematically precise definition of information relevance. Under this semantic representation framework, they defined the semantic entropy $H_{s}\left(\tilde{X}\right)$, semantic channel capacity $C_{s}$, and semantic rate distortion function $R_{s}\left(D\right)$. Building on this foundation, recent works have extended SIT to semantic arithmetic coding [29], semantic rate distortion optimization [30], semantic Markov chains [31], and compression with side information [32]. While SIT establishes a rigorous mathematical foundation, the existing capacity analysis within the SIT framework mainly focuses on AWGN channels, and its extension to wireless fading channels is still lacking.

This paper investigates the semantic channel capacity of Rayleigh fading channels under synonymous mapping. We first derive the semantic channel capacity when synonymous mapping is applied to the channel fading coefficient *h*. By partitioning the channel gain into *K* synonymous intervals, the semantic capacity formula $C_{s}^{\left(h\right)}=\sum_{i=1}^{K}\pi_{i}\log_{2}\left(1+\frac{P}{N_{0}}g_{i}\right)$ is established, where $\pi_{i}$ and $g_{i}$ denote the probability and the conditional expectation of ${\left|h\right|}^{2}$ within the *i*th synonymous interval, respectively. The corresponding theoretical upper and lower bounds are also derived using Jensen’s inequality. To determine an optimized synonymous partition, we formulate the partitioning problem as a (K−1)-dimensional nonlinear constrained optimization problem and employ a neural network-based approach with the Adam optimizer [33] to obtain numerical solutions. Furthermore, we extend the framework by applying synonymous mapping to both the channel fading coefficient and the additive noise. Under the independence assumption, the semantic capacity formula $C_{s}^{\left(h,n\right)}=\sum_{i=1}^{K}\sum_{j=1}^{J}\pi_{i}\pi_{j}\log_{2}\left(1+\frac{P}{v_{j}}g_{i}\right)$ and its upper and lower bounds are derived, where $\pi_{j}$ and $v_{j}$ represent the probability and conditional variance of noise within the *j*th noise synonymous interval, respectively. Our numerical results illustrate the theoretical semantic channel capacity under synonymous mapping, validate the theoretical analysis, and demonstrate the compatibility of the proposed framework with both CIT and SIT.

The remainder of this paper is organized as follows. Section 2 introduces the notation conventions, synonymous mapping, and Rayleigh fading channel model. Section 3 derives the semantic capacity under channel fading coefficient synonymous mapping and develops the neural network-based optimization framework. Section 4 extends the analysis to synonymous mapping of both channel gain and noise. Section 5 provides numerical results and analysis. Section 6 concludes the paper and discusses future research directions.

## 2. System Model and Preliminaries

### 2.1. Notation and Conventions

Throughout this paper, random variables are represented by uppercase letters (e.g., *X* or *Y*), with their realizations denoted by the corresponding lowercase letters (e.g., *x* or *y*). Sets are indicated by calligraphic letters (e.g., X or Y). Semantic random variables and their alphabets are distinguished by a tilde notation, such as $\tilde{X}$ or $\tilde{X}$. We denote p(·) as the probability density function (PDF), while q(·) and Q(·) represent the PDF and cumulative distribution function (CDF) of the standard normal distribution, respectively. The expectation is denoted by E[·], log(·) denotes the natural logarithm, and $\log_{2}\left(\cdot \right)$ denotes the base-2 logarithm. Let f:X→Y denote a mapping from set X to set Y.

### 2.2. Synonymous Mapping

In semantic communication, the notion of meaning of information plays a central role and has been widely discussed in both natural language and artificial intelligence [34,35,36,37]. In this paper, we adopt the following task-oriented characterization of the meaning of information.

**Definition** **1** (Task-Oriented Meaning of Information)**.** *The meaning of an information symbol is characterized in a task-oriented manner as the equivalence class of all syntactic representations that are equally relevant and important to the receiver for the considered task. Two symbols are said to carry the same meaning if and only if they belong to the same synonymous class, i.e., they produce equivalent outcomes at the receiver for the considered task.*

This characterization is consistent with the semantic variable in SIT [28] and is supported by well-established examples. In WordNet [34], words such as “happy”, “joyful”, and “content” form one synonym set because they produce equivalent outcomes in the reader’s understanding. Similarly, in deep learning-based classifiers, syntactically diverse inputs, such as different images of the same object under varying viewpoints or lighting, are mapped to the same semantic label [37]. This is because they are equally relevant and important for the classification task and therefore share the same meaning. In the SIT framework, this equivalence is formally introduced through the concept of synonymous mapping. In the present context, semantic equivalence is characterized over the sample space of individual realizations for the considered task. Sequence-level or time-dependent semantic representations [38] can be incorporated by extending the present single-sample formulation to a sequence-based one.

Following the natural evolution of CIT from discrete to continuous domains, SIT provides a rigorous mathematical extension for continuous sources in Section IX [28]. For a continuous random variable *X* with PDF p(x), the synonymous mapping framework is defined according to Definition 26 in [28]. Let Ω⊂R denote the continuous support set of *X*. Let $\tilde{X}$ be the associated discrete semantic variable with a semantic alphabet $\tilde{X}=\left\{{\tilde{x}}_{1},\ldots ,{\tilde{x}}_{\tilde{N}}\right\}$, where each semantic symbol ${\tilde{x}}_{i}$ represents one distinct meaning. The synonymous mapping is defined as follows:

(1) $$f_{x}:\tilde{X}\to \Omega ,$$

which partitions Ω into $\tilde{N}$ disjoint synonymous intervals, i.e., $\Omega ={⋃}_{i=1}^{\tilde{N}}\Omega_{i}$ and $\Omega_{i}\cap \Omega_{j}=\emptyset$ for all i≠j. Each semantic symbol ${\tilde{x}}_{i}$ corresponds to one synonymous interval $\Omega_{i}\subset \Omega$. Under the mapping $f_{x}$, all syntactic realizations within the same interval $\Omega_{i}$ are semantically equivalent and represented by the semantic symbol ${\tilde{x}}_{i}$. The semantic probability for each interval is

(2) $$P_{s}\left({\tilde{x}}_{i}\right)=\int_{\Omega_{i}}p\left(x\right)dx.$$

**Figure 1:**
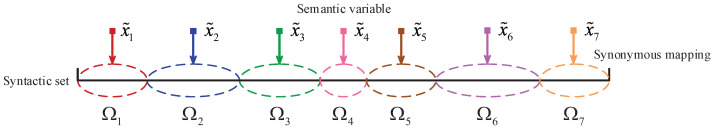
An example of synonymous mapping for a continuous random variable.

As illustrated in Figure 1, each semantic symbol corresponds to a synonymous interval of syntactic realizations.

Under the synonymous mapping framework, reliable communication is judged at the semantic symbol level rather than the syntactic symbol level. Let *X* and $\hat{X}$ denote the transmitted and recovered syntactic symbols, respectively. Let $\tilde{X}$ and $\hat{\tilde{X}}$ denote the transmitted and recovered semantic symbols, respectively. Semantic decoding is regarded as successful if and only if

(3) $$\hat{\tilde{X}}=\tilde{X},$$

i.e., *X* and $\hat{X}$ belong to the same synonymous class. Accordingly, the semantic error probability is defined as follows:

(4) $$P_{e}^{\left(s\right)}=\mathrm{Pr}\left(\hat{\tilde{X}}\neq \tilde{X}\right).$$

**Remark** **1.** *Under this criterion, the semantic channel capacity studied in this paper should be interpreted operationally as a task-dependent achievable rate under semantic equivalence constraints induced by synonymous mapping, rather than the Shannon capacity under the classical exact syntactic symbol recovery criterion. For consistency with the SIT literature, we retain the term **semantic channel capacity** in this paper.*

### 2.3. System Model

**Figure 2:**
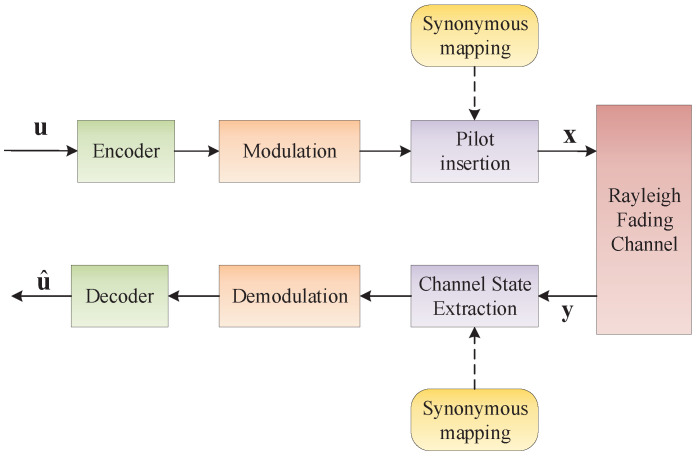
Block diagram of semantic communication system over Rayleigh fading channel.

Based on the foundational concept of synonymous mapping, Figure 2 illustrates the complete system architecture over a Rayleigh fading channel. The source information u is processed by the encoder to produce the transmitted signal x, which satisfies the power constraint $E[|x{|}^{2}]\leq P$. At the transmitter, the pilot insertion block is designed under the synonymous mapping framework. The signal then passes through a Rayleigh fading channel, where the received signal is modeled as follows:

(5) y=hx+n,

where *h* is the channel fading coefficient following a Rayleigh distribution and $n∼N(0,N_{0})$ is the additive white Gaussian noise (AWGN). For Rayleigh fading, |h| has the PDF

(6) $$p_{\left|h\right|}\left(r\right)=\frac{r}{\sigma^{2}}e^{-\frac{r^{2}}{2\sigma^{2}}},\;r\geq 0.$$

By variable transformation, the channel power gain ${\left|h\right|}^{2}$ follows an exponential distribution with the PDF

(7) $$p_{{\left|h\right|}^{2}}\left(r\right)=\frac{1}{2\sigma^{2}}e^{-\frac{r}{2\sigma^{2}}},\;r\geq 0.$$

Without loss of generality, we normalize the channel gain throughout this paper such that $E[|h{|}^{2}]=1$. The ergodic capacity in classical information theory is then given by [39]

(8) $$C=E_{h}\left[\log_{2}\left(1+\gamma |h{|}^{2}\right)\right]=\int_{0}^{\infty }\log_{2}\left(1+\gamma x\right)e^{-x}dx,$$

where $\gamma =P/N_{0}$ is the average signal-to-noise ratio (SNR).

At the receiver, the channel state extraction module estimates the channel state guided by the synonymous mapping, which partitions the continuous channel power gain ${\left|h\right|}^{2}$ into discrete semantic representatives. Subsequently, signal detection is performed based on the noise synonymous mapping. This mapping analogously partitions noise realizations into semantic equivalence classes. Finally, the decoder recovers the source information $\hat{u}$. It is worth noting that, consistent with SIT, this paper focuses on the theoretical derivation of the semantic channel capacity and does not depend on specific encoder or decoder designs or implementation methods. The detailed theoretical analysis of synonymous mappings is presented in Section 3 and Section 4, respectively.

## 3. Semantic Capacity Under Synonymous Mapping of ${\left|h\right|}^{2}$

In this section, we derive the semantic channel capacity under synonymous mapping of the channel fading coefficient *h*. Specifically, we define the synonymous intervals and their semantic representatives, then derive the semantic capacity formula, and finally investigate optimized synonymous partitioning under the given constraints.

### 3.1. Synonymous Mapping and Semantic Representatives

Unlike traditional systems that require precise channel state information (CSI), semantic communication can exploit tolerance to fading variations by treating different channel states as synonymously equivalent. This reduces CSI estimation overhead while preserving semantic fidelity. For the sake of analytical tractability, we introduce the synonymous mapping of the channel power gain ${\left|h\right|}^{2}$. This approach is motivated by two considerations: (1) ${\left|h\right|}^{2}$ follows a simple exponential distribution that facilitates theoretical derivation, and (2) the channel capacity is directly determined by ${\left|h\right|}^{2}$ rather than *h* itself. Therefore, following the SIT framework [28], the continuous range [0,∞) of ${\left|h\right|}^{2}$ is partitioned into *K* disjoint synonymous intervals, where all realizations of ${\left|h\right|}^{2}$ within the same interval are considered semantically equivalent. The *i*th synonymous interval for ${\left|h\right|}^{2}$ is defined by

(9) $$S_{i}=\left[t_{i-1},t_{i}\right),\;i=1,2,\ldots ,K,$$

where $0=t_{0}<t_{1}<t_{2}<\cdots <t_{K-1}<t_{K}=\infty$ are the interval boundaries. To characterize each synonymous interval, we use the conditional expectation as the semantic representative. Specifically, for interval $S_{i}$, the semantic representative $g_{i}$ is defined as follows:

(10) $$g_{i}={E[|h|}^{2}|{\left|h\right|}^{2}\in S_{i}]=\frac{1}{\pi_{i}}\int_{t_{i-1}}^{t_{i}}xe^{-x}dx,\;i=1,2,\ldots ,K,$$

where $\pi_{i}=e^{-t_{i-1}}-e^{-t_{i}}$ is the probability of synonymous interval $S_{i}$. By evaluating the integral, we obtain the closed-form expression

(11) $$g_{i}=\frac{\left(t_{i-1}+1\right)e^{-t_{i-1}}-\left(t_{i}+1\right)e^{-t_{i}}}{e^{-t_{i-1}}-e^{-t_{i}}}.$$

It is worth noting that the above approach can also be interpreted from a quantization perspective. Synonymous mapping is essentially a quantization operation on the continuous channel gain ${\left|h\right|}^{2}$, mapping from the continuous space to a *K*-dimensional discrete semantic space. The choice of conditional expectation as the quantization representative follows the minimum mean square error (MMSE) principle in classical quantization theory, ensuring minimal quantization distortion. From this viewpoint, the semantic channel capacity can be understood as the achievable communication rate under quantized CSI.

### 3.2. Semantic Channel Capacity

With the semantic representatives established, the semantic channel capacity is now derived. Under the synonymous mapping framework, the channel is characterized by discrete semantic representatives $\left\{g_{1},g_{2},\ldots ,g_{K}\right\}$ instead of continuous channel gain realizations. Consequently, the semantic capacity is obtained by summing the instantaneous capacity weighted by the probability of each semantic representative.

**Theorem** **1.** *For a Rayleigh fading channel with channel gain ${\left|h\right|}^{2}∼\exp\left(1\right)$ and synonymous interval partitioning $\left\{S_{1},\ldots ,S_{K}\right\}$, the semantic channel capacity is given by the following (The unit “sebit” is defined in SIT as the measure for the uncertainty of semantic information.):*

(12) $$C_{s}^{\left(h\right)}=\sum_{i=1}^{K}\pi_{i}\log_{2}\left(1+\gamma g_{i}\right)\;\mathrm{sebits}/s/\mathrm{Hz},$$

*where $\gamma =P/N_{0}$ is the average SNR. The lower and upper bounds are*

(13) $$\log_{2}\left(1+\gamma \prod_{i=1}^{K}g_{i}^{\pi_{i}}\right)\leq C_{s}^{\left(h\right)}\leq \log_{2}\left(1+\gamma \sum_{i=1}^{K}\pi_{i}g_{i}\right),$$

*where $\sum_{i=1}^{K}\pi_{i}g_{i}$ denotes the average gain of the synonymous intervals (arithmetic mean of the semantic representatives) and $\prod_{i=1}^{K}g_{i}^{\pi_{i}}$ is the weighted geometric mean.*

**Proof.**  Let $G_{s}$ denote the discrete semantic channel gain representative induced by the synonymous mapping, which takes values $\{g_{i}{\}}_{i=1}^{K}$ with probabilities ${\left\{\pi_{i}\right\}}_{i=1}^{K}$. The semantic capacity formula is obtained by averaging the instantaneous capacity $\log_{2}\left(1+\gamma G_{s}\right)$ over the discrete semantic channel states; in other words, it is written as

(14) $$C_{s}^{\left(h\right)}=E_{G_{s}}\left[\log_{2}\left(1+\gamma G_{s}\right)\right]=\sum_{i=1}^{K}\pi_{i}\log_{2}\left(1+\gamma g_{i}\right).$$

The achievability of $C_{s}^{\left(h\right)}$ can be proven by extending the Gaussian Semantic Channel Coding Theorem from the SIT framework [28] to the ergodic fading scenario. The detailed proof follows the SIT coding theorem analogously and is omitted here for brevity.

Then, we prove the lower bound using Jensen’s inequality. Since $\log_{2}\left(1+e^{x}\right)$ is convex in *x*, under Jensen’s inequality, we have

(15) $$\begin{array}{cc} C_{s}^{\left(h\right)} & =\sum_{i=1}^{K}\pi_{i}\log_{2}\left(1+e^{\ln(\gamma g_{i})}\right)\\ \; & \geq \log_{2}\left(1+e^{\sum_{i=1}^{K}\pi_{i}\ln\left(\gamma g_{i}\right)}\right)\\ \; & =\log_{2}\left(1+\gamma \prod_{i=1}^{K}g_{i}^{\pi_{i}}\right) \end{array}$$

and complete the proof of the lower bound. Similarly, for the upper bound, Jensen’s inequality for the concave function $\log_{2}\left(\cdot \right)$ yields

(16) $$C_{s}^{\left(h\right)}\leq \log_{2}\left(1+\gamma \sum_{i=1}^{K}\pi_{i}g_{i}\right)\overset{\left(a\right)}{=}\log_{2}\left(1+\gamma \right),$$

where equality (a) holds because the average gain of the synonymous intervals satisfies $\sum_{i=1}^{K}\pi_{i}g_{i}={E[|h|}^{2}]=1$ for the normalized Rayleigh fading channel. Therefore, the upper bound corresponds to the AWGN channel capacity with an SNR γ. □

The established bounds in Equation (13) provide theoretical guarantees for the semantic channel capacity. According to the convexity property of semantic capacity in SIT [28], the relation $C_{s}^{\left(h\right)}\geq C$ holds under synonymous mapping. Moreover, the value of $C_{s}^{\left(h\right)}$ is determined by the number of synonymous intervals *K*:

- When K=1 (AWGN channel), the semantic capacity degenerates to $C_{s}^{\left(h\right)}=\log_{2}\left(1+\gamma \right)$, which achieves the upper bound (AWGN capacity).
- As K→∞ (syntactic Rayleigh channel), the semantic representatives $\left\{g_{i}\right\}$ approach the continuous distribution of ${\left|h\right|}^{2}$, and thus $C_{s}^{\left(h\right)}\to C$.
- For finite *K* (semantic Rayleigh channel), we have $C<C_{s}^{\left(h\right)}<\log_{2}\left(1+\gamma \right)$.

### 3.3. Optimized Synonymous Partitioning with Numerical Constraints

The semantic channel capacity $C_{s}^{\left(h\right)}$ in Equation (12) is determined by the synonymous partitioning, i.e., the synonymous intervals $\left\{S_{1},S_{2},\ldots ,S_{K}\right\}$. Equivalently, it is determined by the interval boundaries $T=\{t_{0},t_{1},t_{2},\ldots ,t_{K-1},t_{K}\}$. To obtain an optimized synonymous partition, we formulate the optimization problem as follows:

(17) $$\begin{array}{cc} \max_{T}\; & C_{s}^{\left(h\right)}\left(T\right)=\sum_{i=1}^{K}\pi_{i}\left(T\right)\log_{2}\left(1+\gamma g_{i}\left(T\right)\right)\\ s.t.\; & 0=t_{0}<t_{1}<t_{2}<\cdots <t_{K-1}<t_{K}=\infty , \end{array}$$

where both $\pi_{i}\left(T\right)$ and $g_{i}\left(T\right)$ are functions of the boundaries T through Equation (10). The problem in Equation (17) is a (K−1)-dimensional constrained nonlinear optimization problem. Theoretical analysis reveals that the objective function $C_{s}^{\left(h\right)}\left(T\right)$ lacks a closed-form expression in terms of T, rendering analytical solutions infeasible. Consequently, a neural network-based approach with the Adam optimizer is adopted as a numerical tool to determine an optimized synonymous partition under the given constraints.

In order to enforce the constraint $0<t_{1}<t_{2}<\cdots <t_{K-1}$, we employ an indirect parameterization. Instead of optimizing T directly, we introduce learnable weights $Z=\left\{z_{1},z_{2},\ldots ,z_{K}\right\}\in R^{K}$ that determine the length of each synonymous interval. Specifically, the weights Z act as learnable priorities that distribute the remaining available range among intervals after assigning a baseline length to all *K* intervals. The interval lengths $\left\{\ell_{1},\ell_{2},\ldots ,\ell_{K}\right\}$ are constrained to lie within $\left[\ell_{\min},\ell_{\max}\right]$ to ensure balanced partitioning and avoid degenerate solutions in which one interval dominates while the others vanish. Such a degenerate case corresponds to the AWGN capacity’s upper bound. In practice, the bounds $\ell_{\min}$ and $\ell_{\max}$ should be selected based on specific task objectives and physical significance to ensure meaningful partitioning. The boundaries are then computed as follows:

(18) $$t_{i}=\sum_{j=1}^{i}\ell_{j},\;i=1,2,\ldots ,K-1,$$

which automatically satisfies the ordering constraint. The mapping from Z to *ℓ* ensures that each interval length lies within the specified bounds through a constrained allocation mechanism. For robustness across different SNR regimes, SNR values are sampled uniformly from a specified range $\left[\gamma_{\min},\gamma_{\max}\right]$ in each training iteration, and the average semantic capacity is optimized:

(19) $$\max_{Z}\;E_{\gamma ∼U(\gamma_{\min},\gamma_{\max})}\left[C_{s}^{\left(h\right)}\left(T\left(Z\right),\gamma \right)\right].$$

The optimization procedure proceeds as follows. First, the boundaries are initialized using equal-probability partitioning, where $t_{i}^{\left(0\right)}=-\ln\left(1-i/K\right)$, which ensures uniform probability across all intervals. A cosine annealing schedule is then applied to the learning rate to facilitate convergence. After convergence, an optimized synonymous partition under the given constraints is obtained, as illustrated in Section 5. It is worth noting that the problem in Equation (17) is generally non-convex, and the proposed Adam-based numerical procedure is used to obtain an optimized synonymous partition under the given constraints rather than to guarantee a globally optimal solution.

## 4. Semantic Capacity Under Both Synonymous Mapping of ${\left|h\right|}^{2}$ and Synonymous Mapping of n

In this section, we further extend synonymous mapping to the additive noise, exploiting the robustness of semantic information to minor perturbations and thereby achieving a more complete characterization of the semantic capacity. Under the independence assumption between *h* and *n*, we first introduce the noise model and its synonymous mapping representation and then derive the semantic channel capacity.

### 4.1. Noise Model and Synonymous Mapping

Recall that the additive noise in Equation (5) follows a Gaussian distribution $n∼N(0,N_{0})$. For notational convenience in the derivations that follow, we denote $\sigma_{n}=\sqrt{N_{0}}$, so that $n∼N(0,\sigma_{n}^{2})$. The noise PDF is given by

(20) $$p\left(n\right)=\frac{1}{\sqrt{2\pi \sigma_{n}^{2}}}\exp\left(-\frac{n^{2}}{2\sigma_{n}^{2}}\right)=\frac{1}{\sigma_{n}}q\left(\frac{n}{\sigma_{n}}\right),$$

where $q\left(u\right)=\frac{1}{\sqrt{2\pi }}e^{-u^{2}/2}$ is the standard normal PDF and $Q\left(u\right)=\int_{-\infty }^{u}q\left(t\right)dt$ is the standard normal CDF.

Similar to the synonymous mapping applied to the channel gain, we partition the noise space into *J* synonymous intervals as follows:

(21) $$L_{j}=\left[b_{j-1},b_{j}\right),\;j=1,2,\ldots ,J,$$

where $-\infty =b_{0}<b_{1}<b_{2}<\cdots <b_{J-1}<b_{J}=\infty$ are the interval boundaries and the probability of synonymous interval $L_{j}$ is given by

(22) $$\pi_{j}=Q\left(\frac{b_{j}}{\sigma_{n}}\right)-Q\left(\frac{b_{j-1}}{\sigma_{n}}\right).$$

As in the channel gain case, the above noise mapping can also be interpreted from a quantization perspective.

**Lemma** **1.** *For Gaussian noise $n∼N(0,\sigma_{n}^{2})$ with a synonymous interval $L_{j}=\left[b_{j-1},b_{j}\right)$ and normalized boundaries $\alpha =b_{j-1}/\sigma_{n}$, $\beta =b_{j}/\sigma_{n}$, the semantic representatives (conditional mean and variance) are*

(23) $$\begin{array}{cc} \mu_{j} & =\sigma_{n}\frac{q(\alpha )-q(\beta )}{Q(\beta )-Q(\alpha )}, \end{array}$$

(24) $$\begin{array}{cc} v_{j} & =\sigma_{n}^{2}\left\{1-\frac{\beta q(\beta )-\alpha q(\alpha )}{Q(\beta )-Q(\alpha )}-{\left[\frac{q(\alpha )-q(\beta )}{Q(\beta )-Q(\alpha )}\right]}^{2}\right\}. \end{array}$$

**Proof.**  Let $\alpha =b_{j-1}/\sigma_{n}$ and $\beta =b_{j}/\sigma_{n}$. The conditional mean is defined as follows:

(25) $$\mu_{j}=E\left[n|L_{j}\right]=\frac{\int_{L_{j}}np\left(n\right)dn}{\pi_{j}}=\frac{\sigma_{n}\int_{\alpha }^{\beta }uq\left(u\right)du}{Q(\beta )-Q(\alpha )}.$$

Using the identity $q^{\prime }\left(u\right)=-uq\left(u\right)$, we can obtain

(26) $$\int_{\alpha }^{\beta }uq\left(u\right)du=-\int_{\alpha }^{\beta }q^{\prime }\left(u\right)du=q\left(\alpha \right)-q\left(\beta \right).$$

By substituting this result into Equation (25), $\mu_{j}$ can be rewritten as

(27) $$\mu_{j}=\sigma_{n}\frac{q(\alpha )-q(\beta )}{Q(\beta )-Q(\alpha )}$$

Next, we derive the conditional variance. By the definition of variance, we have

(28) $$\begin{array}{cc} v_{j} & =\mathrm{Var}(n|L_{j})\\ \; & =E\left[n^{2}|L_{j}\right]-\mu_{j}^{2}\\ \; & =\frac{\int_{L_{j}}n^{2}p\left(n\right)dn}{\pi_{j}}-\sigma_{n}^{2}{\left[\frac{q(\alpha )-q(\beta )}{Q(\beta )-Q(\alpha )}\right]}^{2}\\ \; & =\frac{\int_{b_{j-1}}^{b_{j}}n^{2}\frac{1}{\sigma_{n}}q\left(\frac{n}{\sigma_{n}}\right)dn}{Q(\beta )-Q(\alpha )}-\sigma_{n}^{2}{\left[\frac{q(\alpha )-q(\beta )}{Q(\beta )-Q(\alpha )}\right]}^{2} \end{array}$$

With the substitution $u=n/\sigma_{n}$, the integral becomes

(29) $$\int_{b_{j-1}}^{b_{j}}n^{2}\frac{1}{\sigma_{n}}q\left(\frac{n}{\sigma_{n}}\right)dn=\sigma_{n}^{2}\int_{\alpha }^{\beta }u^{2}q\left(u\right)du.$$

To evaluate $\int u^{2}q\left(u\right)du$, we note the identity

(30) $$\frac{d}{du}\left[-uq\left(u\right)\right]=-q\left(u\right)+u^{2}q\left(u\right).$$

From this, we obtain

(31) $$u^{2}q\left(u\right)=q\left(u\right)+\frac{d}{du}\left[-uq\left(u\right)\right].$$

Integrating both sides gives

(32) $$\int u^{2}q\left(u\right)du=Q\left(u\right)-uq\left(u\right)+C.$$

Therefore, by combining Equations (29) and (32), Equation (28) can be rewritten as

(33) $$\begin{array}{cc} v_{j} & =\frac{\sigma_{n}^{2}\int_{\alpha }^{\beta }u^{2}q\left(u\right)du}{Q(\beta )-Q(\alpha )}-\sigma_{n}^{2}{\left[\frac{q(\alpha )-q(\beta )}{Q(\beta )-Q(\alpha )}\right]}^{2}\\ \; & =\sigma_{n}^{2}\frac{Q(\beta )-\beta q(\beta )-Q(\alpha )+\alpha q(\alpha )}{Q(\beta )-Q(\alpha )}-\sigma_{n}^{2}{\left[\frac{q(\alpha )-q(\beta )}{Q(\beta )-Q(\alpha )}\right]}^{2}\\ \; & =\sigma_{n}^{2}\left\{1-\frac{\beta q(\beta )-\alpha q(\alpha )}{Q(\beta )-Q(\alpha )}-{\left[\frac{q(\alpha )-q(\beta )}{Q(\beta )-Q(\alpha )}\right]}^{2}\right\}. \end{array}$$

□

These semantic representatives $\left\{\mu_{j},v_{j}\right\}$ characterize the statistical properties of Gaussian noise within each synonymous interval, which will be used to derive the semantic channel capacity in the next subsection.

### 4.2. Semantic Channel Capacity

In this subsection, we derive the semantic channel capacity when both channel gain and noise are partitioned into synonymous intervals. Due to the independence between *h* and *n*, there are K×J semantic representative pairs, indexed by (i,j) with i=1,…,K and j=1,…,J. Each pair $\left(g_{i},v_{j}\right)$ has a probability $\pi_{i}\pi_{j}$. The semantic capacity is then obtained by averaging the instantaneous capacity over all semantic representative pairs.

For the theoretical derivation below, we consider an idealized setting in which the semantic noise state is available at the receiver as side information. This assumption does not imply that the noise is independently observable from the received signal; rather, it serves as a benchmark for characterizing the semantic channel capacity under receiver-side semantic noise state information. Under this assumption, the continuous noise realizations are represented by discrete semantic states, and the corresponding expression is obtained by averaging over the joint semantic channel gain and noise state distribution.

**Theorem** **2.** *Consider an idealized setting in which the semantic noise state is available at the receiver as side information. Given K synonymous intervals $\left\{S_{1},\ldots ,S_{K}\right\}$ for channel gain and J synonymous intervals $\left\{L_{1},\ldots ,L_{J}\right\}$ for noise, the semantic channel capacity of a Rayleigh fading channel under this assumption is given by*

(34) $$C_{s}^{\left(h,n\right)}=\sum_{i=1}^{K}\sum_{j=1}^{J}\pi_{i}\pi_{j}\log_{2}\left(1+\frac{P}{v_{j}}g_{i}\right)\;\mathrm{sebits}/s/\mathrm{Hz},$$

*with $\pi_{i}$ and $g_{i}$ defined in Equation* (10) *and $\pi_{j}$ and $v_{j}$ given by Equations* (22) *and* (24)*, respectively. The lower and upper bounds are*

(35) $$C_{s}^{\left(h\right)}=\sum_{i=1}^{K}\pi_{i}\log_{2}\left(1+\frac{P}{\sum_{j=1}^{J}\pi_{j}v_{j}}g_{i}\right)\leq C_{s}^{\left(h,n\right)}\leq \sum_{j=1}^{J}\pi_{j}\log_{2}\left(1+\frac{P}{v_{j}}\sum_{i=1}^{K}\pi_{i}g_{i}\right),$$

*where $C_{s}^{\left(h\right)}$ represents the semantic capacity with only channel gain synonymous mapping from Theorem 1.*

**Proof.**  The semantic capacity formula in Equation (34) follows directly from averaging the instantaneous capacity $\log_{2}\left(1+\frac{P}{v_{j}}g_{i}\right)$ over all semantic representative pairs $\left(g_{i},v_{j}\right)$ with probabilities $\pi_{i}\pi_{j}$. Next, we derive the bounds for $C_{s}^{\left(h,n\right)}$. To establish the lower bound, we apply Jensen’s inequality over the noise dimension while fixing the channel gain index *i*. For a>0, the function $f\left(x\right)=\log_{2}\left(1+\frac{a}{x}\right)$ is convex in x>0. Under Jensen’s inequality, we have

(36) $$\begin{array}{cc} C_{s}^{\left(h,n\right)} & =\sum_{i=1}^{K}\pi_{i}\sum_{j=1}^{J}\pi_{j}\log_{2}\left(1+\frac{P}{v_{j}}g_{i}\right)\\ \; & \geq \sum_{i=1}^{K}\pi_{i}\log_{2}\left(1+\frac{P}{\sum_{j=1}^{J}\pi_{j}v_{j}}g_{i}\right)\\ \; & \overset{\left(a\right)}{=}\sum_{i=1}^{K}\pi_{i}\log_{2}\left(1+\frac{P}{N_{0}}g_{i}\right)\\ \; & =\sum_{i=1}^{K}\pi_{i}\log_{2}\left(1+\gamma g_{i}\right)\\ \; & =C_{s}^{\left(h\right)}, \end{array}$$

where equality (a) holds since $\sum_{j=1}^{J}\pi_{j}v_{j}=N_{0}$. The resulting lower bound is equivalent to $C_{s}^{\left(h\right)}$ from Theorem 1, where only channel gain synonymous mapping is applied. For the upper bound, we apply Jensen’s inequality over the channel gain dimension while fixing the noise index *j*. Since $\log_{2}\left(x\right)$ is concave, we obtain

(37) $$\begin{array}{cc} C_{s}^{\left(h,n\right)} & =\sum_{j=1}^{J}\pi_{j}\sum_{i=1}^{K}\pi_{i}\log_{2}\left(1+\frac{P}{v_{j}}g_{i}\right)\\ \; & \leq \sum_{j=1}^{J}\pi_{j}\log_{2}\left(\sum_{i=1}^{K}\pi_{i}\left(1+\frac{P}{v_{j}}g_{i}\right)\right)\\ \; & =\sum_{j=1}^{J}\pi_{j}\log_{2}\left(1+\frac{P}{v_{j}}\sum_{i=1}^{K}\pi_{i}g_{i}\right)\\ \; & \overset{\left(b\right)}{=}\sum_{j=1}^{J}\pi_{j}\log_{2}\left(1+\frac{P}{v_{j}}\right), \end{array}$$

where equality (b) follows from $\sum_{i=1}^{K}\pi_{i}g_{i}={E[|h|}^{2}]=1$. Thus, we complete the proof. □

Theorem 2 extends the semantic capacity framework by incorporating synonymous mappings for both channel gain and noise, yielding a higher capacity $C_{s}^{\left(h,n\right)}\geq C_{s}^{\left(h\right)}$. Beyond this capacity formula, the derived bounds in Equation (35) provide both theoretical insights and practical guidance for system design. Specifically, the lower bound coincides with $C_{s}^{\left(h\right)}$ from Theorem 1, indicating that the effects of channel gain and noise synonymous mappings are cumulative, with noise synonymous mapping providing additional improvement beyond channel gain mapping alone. The upper bound, on the other hand, characterizes the achievable capacity when the conditional noise variance $v_{j}$ is precisely represented for each semantic interval.

### 4.3. Consistency with General SIT Framework

To further validate the theoretical consistency of the proposed framework, we investigate the semantic capacity in a degenerate case where the Rayleigh fading channel reduces to an AWGN channel. We then analyze its compatibility with the foundational SIT theory. Consider the special case where h≡1, such that the PDFs of *h* and ${\left|h\right|}^{2}$ are characterized by the Dirac delta function δ(x−1). Consequently, for any synonymous partitioning $\left\{S_{1},S_{2},\ldots ,S_{K}\right\}$, only one specific interval $S_{k}$ contains the value of one, resulting in

(38) $$\pi_{i}=\left\{\begin{array}{cc} 1, & i=k\\ 0, & i\neq k \end{array}\right..$$

The corresponding semantic representative becomes $g_{k}=1$, and the semantic capacity formula $C_{s}^{\left(h\right)}$ simplifies directly to

(39) $$C_{s}^{\left(h\right)}=\log_{2}\left(1+\gamma \right),$$

which indicates that the semantic channel capacity converges to the upper bound established in Theorem 1. Regarding the semantic capacity $C_{s}^{\left(h,n\right)}$, when h≡1, the expression is given by

(40) $$C_{s}^{\left(h,n\right)}=\sum_{j=1}^{J}\pi_{j}\log_{2}\left(1+\frac{P}{v_{j}}\right)$$

By defining the term $N_{0}/v_{j}$ as the synonymous length (consistent with the SIT framework [28]) and applying the property $\log_{2}\left(1+\gamma \cdot \frac{N_{0}}{v_{j}}\right)\leq \log_{2}\left(1+\gamma \right)+\log_{2}\left(\frac{N_{0}}{v_{j}}\right)$ for $N_{0}/v_{j}\geq 1$, we obtain

(41) $$\begin{array}{cc} C_{s}^{\left(h,n\right)} & =\sum_{j=1}^{J}\pi_{j}\log_{2}\left(1+\frac{P}{N_{0}}\cdot \frac{N_{0}}{v_{j}}\right)\\ \; & \leq \log_{2}\left(1+\gamma \right)+\sum_{j=1}^{J}\pi_{j}\log_{2}\left(\frac{N_{0}}{v_{j}}\right)\\ \; & =\log_{2}\left(1+\gamma \right)+E\left[\log_{2}\left(\frac{N_{0}}{v_{j}}\right)\right]. \end{array}$$

The expression in Equation (41) is mathematically equivalent to the general semantic capacity derived in [28], thereby ensuring that our proposed framework remains a valid generalization of SIT under channel degradation.

## 5. Numerical Results

In this section, we present the numerical results to evaluate the semantic channel capacity over Rayleigh fading channels. To provide a comprehensive benchmark, we introduced the classical Rayleigh capacity *C* and the AWGN channel capacity $C_{AWGN}$ as baselines. The semantic capacity was calculated directly from the closed-form expressions derived in Theorems 1 and 2. Since the channel gain ${\left|h\right|}^{2}$ followed an exponential distribution over [0,∞), we imposed a practical threshold ${\left|h\right|}^{2}\leq 10$ for numerical evaluation, as the probability of exceeding this threshold was less than 0.005% and contributed negligibly to the capacity. It should be noted that the classical capacity was measured in bits/s/Hz, whereas the semantic capacity was measured in sebits/s/Hz. In the SIT framework, a sebit measures the uncertainty of semantic symbols, where each semantic symbol corresponds to a synonymous set of syntactic realizations induced by the synonymous mapping. From this perspective, the classical bit-based description can be viewed as a special case in which each synonymous set contains only one syntactic realization. Therefore, the comparisons in this paper are presented to illustrate the theoretical semantic capacity results and to demonstrate the compatibility of the proposed framework with CIT.

**Figure 3:**
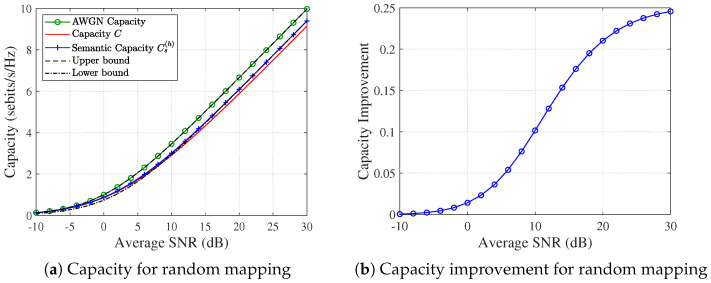
Semantic capacity $C_{s}^{\left(h\right)}$ and classic capacity *C* of Rayleigh fading channel (K=8).

Figure 3a presents the capacity *C*, the semantic capacity $C_{s}^{\left(h\right)}$ under random synonymous mapping, and the corresponding theoretical upper and lower bounds derived in Theorem 1. The number of synonymous intervals was set to K=8, and the SNR ranged from −10 dB to 30 dB. The numerical results show that $C_{s}^{\left(h\right)}$ was larger than *C* across all SNR regimes, which is consistent with the property of semantic capacity in SIT [28]. The simulated values lied within the theoretical bounds, validating the analytical derivations. As shown in Figure 3b, the capacity improvement increased with the SNR, reaching approximately 0.25 sebits/s/Hz at 30 dB. The gap between $C_{s}^{\left(h\right)}$ and the upper bound indicates that further capacity gain can be achieved through optimization of the synonymous intervals.

**Figure 4:**
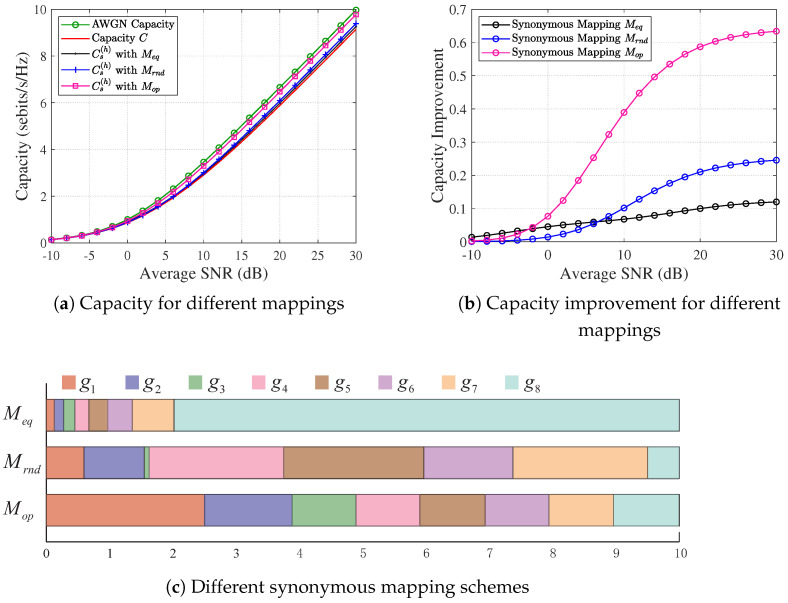
Comparison of different synonymous mapping strategies (K=8).

For the neural network-based optimization of interval boundaries, we employed the Adam optimizer with a cosine annealing learning rate schedule over 2000 training epochs. For the optimization, we enforced practical constraints on the interval lengths; each synonymous interval $S_{i}$ had to satisfy $\ell_{\min}=5/K\leq \ell_{i}\leq \ell_{\max}=20/K$, where $\ell_{i}=t_{i}-t_{i-1}$. These values were chosen heuristically based on the truncated channel gain range ${\left|h\right|}^{2}\in \left[0,10\right]$. They correspond to approximately one half and two times the equal-length partition size, respectively, and helped avoid excessively narrow or wide intervals during optimization. Figure 4 compares the performance of three synonymous mapping strategies with K=8: equal-probability mapping $M_{eq}$, random mapping $M_{rnd}$, and optimized mapping $M_{op}$. As shown in Figure 4b, for all three mappings, the improvement in capacity increased with the SNR. At moderate-to-high SNRs, the semantic capacity result under $M_{op}$ was closest to the upper bound among the three strategies. At 30 dB, $M_{op}$ reached an improvement of approximately 0.63 sebits/s/Hz, compared with 0.25 sebits/s/Hz for $M_{rnd}$ and 0.12 sebits/s/Hz for $M_{eq}$. It should be noted that at low SNRs (below −3 dB), the semantic capacity under $M_{op}$ was slightly lower than that under $M_{rnd}$. This can be attributed to the fact that at a low SNR, the capacity function $\log_{2}\left(1+\gamma g_{i}\right)$ was nearly linear in $g_{i}$, limiting the benefit of neural network-based optimization in refining the interval boundaries. Figure 4c visualizes the specific interval boundaries for the three synonymous mapping schemes. While $M_{eq}$ concentrated intervals in the low channel-gain regime due to the exponential distribution, $M_{op}$ yielded a more balanced partitioning and led to a larger semantic capacity.

**Figure 5:**
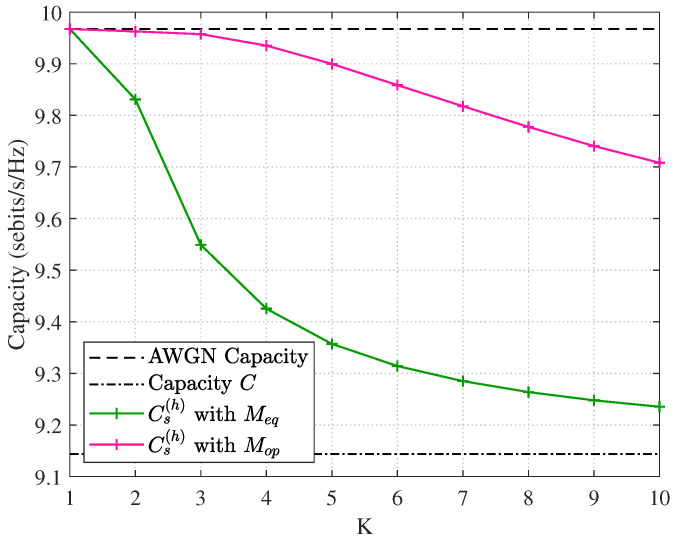
Semantic capacity $C_{s}^{\left(h\right)}$ for different values of *K* (SNR = 30 dB).

Figure 5 illustrates the impact of the number of synonymous intervals *K* on the semantic capacity $C_{s}^{\left(h\right)}$ at SNR = 30 dB. Both equal-probability mapping $M_{eq}$ and optimized mapping $M_{op}$ were evaluated. According to Theorem 1, when K=1, there is only one semantic interval covering the entire channel gain range, and the semantic capacity reaches the AWGN capacity upper bound $\log_{2}\left(1+\gamma \right)\approx 9.97$ sebits/s/Hz. As *K* increases, the semantic capacity monotonically decreases, converging toward the capacity C≈9.15 sebits/s/Hz as K→∞. The numerical results are consistent with this theoretical analysis. For $M_{op}$ and $M_{eq}$, the capacity decreased from approximately 9.97 sebits/s/Hz at K=1 to 9.71 and 9.23 sebits/s/Hz at K=10, respectively.

**Figure 6:**
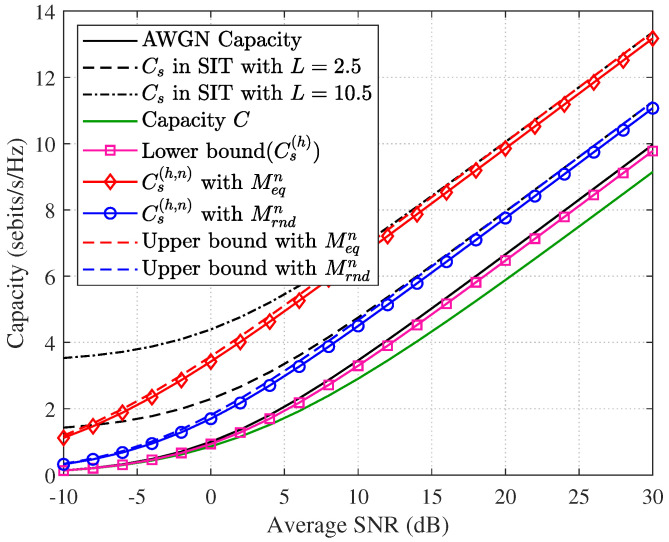
Semantic capacities with different noise synonymous mapping strategies (K=8, J=4).

Figure 6 illustrates the semantic capacity $C_{s}^{\left(h,n\right)}$ when synonymous mapping was applied to both channel gain and noise. We set K=8 synonymous intervals for channel gain and J=4 intervals for noise, resulting in K×J=32 semantic representative pairs $\left(g_{i},v_{j}\right)$. For the channel gain, we employed the neural network-based optimized mapping $M_{op}$ as described in Figure 4, which provided an optimized synonymous partition under the given constraints. For the noise, we considered two synonymous mapping strategies: equal-probability mapping $M_{eq}^{\left(n\right)}$ and random mapping $M_{rnd}^{\left(n\right)}$. Notably, $M_{eq}^{\left(n\right)}$ was adopted here as the optimal partitioning strategy for the noise, as SIT has demonstrated that equal-probability mapping maximizes the semantic capacity for AWGN channels. Given the independence between *h* and *n*, $M_{eq}^{\left(n\right)}$ for noise was effectively equivalent to $M_{op}^{\left(n\right)}$. To demonstrate the compatibility with foundational SIT, we also plotted the theoretical semantic capacity bounds derived from [28] for the AWGN case, denoted as $C_{s}=\log_{2}\left(1+\gamma \right)+\log_{2}\left(L\right)$, where *L* represents the average synonymous length. As shown in Figure 6, both $C_{s}^{\left(h,n\right)}$ with $M_{eq}^{\left(n\right)}$ and $M_{rnd}^{\left(n\right)}$ were consistently larger than $C_{s}^{\left(h\right)}$ and *C* across all SNR values ranging from −10 dB to 30 dB, validating Theorem 2. The theoretical bounds derived in Equation (35) were also plotted, and the simulated semantic capacities lied within the predicted range. Furthermore, the simulated $C_{s}^{\left(h,n\right)}$ values exhibited the same growth trend as the SIT curves, particularly at high SNRs, which demonstrates the compatibility of the proposed framework with the general SIT theory. At a 20-dB SNR, $C_{s}^{\left(h,n\right)}$ with $M_{eq}^{\left(n\right)}$ achieved approximately 9.86 sebits/s/Hz, while $M_{rnd}^{\left(n\right)}$ achieved 7.76 sebits/s/Hz, compared with 6.47 sebits/s/Hz for $C_{s}^{\left(h\right)}$. Relative to the $C_{s}^{\left(h\right)}$, the corresponding increases were 52.4% and 19.9%, respectively. The results demonstrate that $M_{eq}^{\left(n\right)}$ generally outperformed $M_{rnd}^{\left(n\right)}$, particularly at moderate-to-high SNRs, indicating that uniform distribution of noise intervals should be prioritized in the design of semantic communication systems.

It is worth emphasizing that the larger semantic capacity values reported here do not imply a violation of the Shannon limit under the classical syntactic accuracy. Rather, they reflect the change in communication criterion introduced by synonymous mapping, under which multiple syntactic realizations that are equivalent for the considered task are represented by the same semantic symbol. Under this semantic fidelity, the receiver no longer requires exact recovery of every syntactic realization, and the resulting semantic channel capacity characterizes the maximum rate of reliable semantic symbol transmission under the given synonymous mapping.

## 6. Conclusions

In this paper, we proposed a semantic channel capacity framework for Rayleigh fading channels based on synonymous mapping. We derived closed-form expressions for the semantic channel capacity under two scenarios: synonymous mapping of channel gain only ($C_{s}^{\left(h\right)}$) and synonymous mapping of both channel gain and noise ($C_{s}^{\left(h,n\right)}$). The corresponding theoretical bounds were also established, showing that the relation $C_{s}^{\left(h,n\right)}\geq C_{s}^{\left(h\right)}\geq C$ held under synonymous mapping, where *C* denotes the classical Rayleigh channel capacity. A neural network-based optimization approach was further developed to determine the interval boundaries under optimized synonymous partitioning. The numerical results validate the theoretical analysis, illustrate the semantic channel capacity under synonymous mapping, and demonstrate the compatibility of the proposed framework with both CIT and SIT.

Future work can extend this framework to other channels (e.g., Nakagami-*m* channel or Rician channel) and investigate their semantic capacity under synonymous mapping. Additionally, the derived capacity limits can guide the design of practical semantic channel coding algorithms to achieve higher communication efficiency.

## Data Availability

The original contributions presented in this study are included in the article. Further inquiries can be directed to the corresponding author.

## Abbreviations

The following abbreviations are used in this manuscript:

AWGN	Additive white Gaussian noise
CIT	Classical information theory
CSI	Channel state information
MMSE	Minimum mean square error
SIT	Semantic information theory
SNR	Signal-to-noise ratio
